# Supplementary material for: Complexome profiling of the Chlamydomonas psb28 mutant reveals TEF5 as an early PSII assembly factor
Source: Plant Cell. 2025 Mar 17;37(6):koaf055. doi: 10.1093/plcell/koaf055 (PMC12164590; doi:10.1093/plcell/koaf055)
Supplement: koaf055_Supplementary_Data [file koaf055_supplementary_data.zip › psb28_interactive.zip!/plots/intensity/qualityPlots/peptides/_Cre03.g183900.t1.2.html]

### Cre03.g183900.t1.2

Trivial name:   
SeqCoverage [median]: 15.60  
Mass: 14.41 kDa  
  
 

| Sequence | MissedCleavage | Mass [Da] | wtIntensity | muIntensity | Score |
| --- | --- | --- | --- | --- | --- |
| MANVPTMADLEK | 0 | 1318.63 | 0.00 | 1965.38 | 81.77 |
| MANVPTMADLEKNDSAPPAAK | 1 | 2170.04 | 22925.32 | 34385.10 | 128.01 |
| NDSAPPAAK | 0 | 869.42 | 0.00 | 494.61 | 64.27 |
|  |  |  | 22925.3 | 36845.1 |  |
